# Education Cost of Dental Students Using the Activity-Based Costing Method at Iran University of Medical Sciences

**DOI:** 10.4314/ejhs.v35i4.8

**Published:** 2025-07

**Authors:** Mahmoud Zamandi, Behzad Raei

**Affiliations:** 1 Iran University of Medical Sciences, Tehran, Iran; 2 Department of Health, Safety, and Environment Management, School of Public Health, Zanjan University of Medical Sciences, Zanjan, Iran

**Keywords:** Dental Education, Costing, ABC, Education Economics

## Abstract

**Background:**

Accurate cost and pricing information is essential for optimal budget allocation and sound strategic decision-making. Establishing an appropriate costing system is critical for understanding educational expenses, improving services, and managing budgets more efficiently. This study aimed to estimate the cost of educating students at the dental school during the 2022-23 academic year.

**Methods:**

This cross-sectional study estimated the cost of educating dental students at Iran University of Medical Sciences in 2023 using the Activity-Based Costing (ABC) method. Data were collected through interviews with financial and administrative officials, as well as relevant units such as accounting and administration, utilizing a structured checklist. After estimating the costs of activity centers, overhead and intermediate center expenses were allocated to final activity centers based on suitable allocation bases. Ultimately, the costs associated with the final activity centers which is, the educational departments, were determined, and the total cost per student was calculated.

**Results:**

The average cost of educating a general dentistry student in the 2022-23 academic year was approximately 132,438 Purchasing Power Parity (PPP) adjusted US Dollars. In the dental school, total overhead costs amounted to 802,953, while total direct costs were 1,133,853 in PPP-adjusted dollars. The largest portion of total costs (61%) was attributed to human resources, followed by consumables (20%) and equipment depreciation (14%).

**Conclusion:**

The majority of costs are allocated to human resources, indicating the need to enhance productivity. Reducing consumable costs and equipment depreciation could further improve financial efficiency. It is recommended that managers and policymakers implement strategies to optimize resources and enhance educational quality.

## Introduction

The increase in the minimum wage and the rise in per capita Gross Domestic Product (GDP) have led to higher tuition and educational costs in dentistry, to the extent that these increases could eventually be passed on to patients, thereby limiting access to dental services for low-income households. In response, governments have decided to provide universities with subsidies to help manage tuition fees (1). In addition to tuition, living expenses and the loss of income during a four- to five-year academic period impose a significant financial burden on students. These costs create a barrier, particularly for low-income groups who are more reliant on loans to continue their education. Overall, costs are a key factor in valuation, and the payment of expenses is a major issue involving all stakeholders, including the university, government, students, professors, staff, patients, and alumni (2). Dental courses are typically among the most expensive educational programs, as they require the provision of specific clinical experiences for students (3).

This study explores the rising costs of dental education and their impact on students, particularly at Iran University of Medical Sciences. The central issue addressed is how escalating tuition, living expenses, and the loss of income during study affect students' financial stability and their ability to enter the dental profession. More specifically, the study aims to estimate the cost of dental education using the Activity-Based Costing (ABC) method, which provides a more accurate measure by identifying various activities involved and assigning associated costs.

Students living in rented accommodations face significantly higher annual living expenses compared to those residing in their family homes. Many students do not have an additional source of income and rely on financial support from their families, indicating a lack of economic and social diversity. Consequently, the high cost of education and associated living expenses can prevent many talented students from low-income families from pursuing further education in the dental field (4). In the United States, tuition fees for dental schools vary considerably, from approximately $20,000 per year at public universities to as much as $90,000 per year at private institutions. The average annual tuition is generally around $50,000, which typically covers only the cost of education, excluding other expenses such as miscellaneous fees, dental equipment, housing, and food, which can total up to $200,000 per year. It was previously believed that the increase in the number of dental graduates and dentists would lead to competition among them, thus driving down the cost of dental services (5).

In the United States, rising educational costs have led to a significant increase in student loan debt, which reached $1.2 trillion by May 2013, making it the second-largest category of consumer debt after mortgages. This escalation in costs and student loan debt remains a major concern for students and their families (6).

The costs of dental education are a significant issue for students, families, universities, and the broader healthcare system. Understanding these costs and their impact on access to dental services is essential for informing policy decisions. As tuition rates and living expenses continue to rise, the importance of this study grows, especially in low-resource settings where these costs may prevent talented students from entering the dental profession. The ABC method provides a detailed and more precise way of understanding educational costs, helping institutions better manage resources.

The link between investing in education and the development of human capital, which drives economic growth, rests on the notion that such investments may not yield immediate returns but will result in both financial and non-financial benefits in the future (7). A cost-benefit analysis study found that the average rate of return on investment in dental education was 29.4%, exceeding the stock market's return rate of 14.35%. The study also revealed that over a 12-year period, the costs of obtaining a dental degree increased at an annual rate of 4.45%, while the average annual income of dentists grew by 3.8% (8). In countries with limited resources and inadequate infrastructure, continuous evaluation of the educational environment's impact on dental education is even more critical. In such contexts, dental educators must adopt student-centered teaching approaches to achieve the best outcomes (9).

Given the increasing costs of dental education and their burden on students, this study is necessary to assess the financial strain placed on students, particularly those from low-income backgrounds. Additionally, in countries with limited educational resources, understanding the impact of educational costs becomes even more urgent. By using the ABC method, this study provides clearer insights into the cost structure of dental education, offering valuable data for policymakers to make informed decisions on subsidies and resource allocation.

Training an optimal number of dentists at a reasonable cost for both students and institutions requires fundamental changes to educational programs. These changes include redesigning the educational process, adjusting the duration of study, and developing curricula tailored to different regions. Without adopting more effective teaching methods, the future of the dental profession may face undesirable outcomes (10). Therefore, those considering dentistry as a career need more comprehensive information, including direct educational costs (e.g., tuition, books, stationery, and dental instruments) and indirect costs (e.g., lost income during the study period).

Cost analysis, as an economic tool in decision-making, can assist managers in improving program effectiveness. By attributing direct and indirect costs, costing helps managers and policymakers determine whether costs exceed total revenues and available subsidies (11). Moreover, unit cost analysis plays a crucial role in planning, budgeting, control, and organizational assessment. By evaluating the performance of cost centers and comparing it with budgeted outcomes, managers can identify areas that need attention and take corrective action (12, 13).

This study's objective was to estimate the cost of educating dental students at Iran University of Medical Sciences for the 2022-23 academic year using the Activity-Based Costing (ABC) method.

## Methods

This applied study estimates the total cost of educating a dental student at Iran University of Medical Sciences (IUMS) during the 2022-23 academic year using the ABC method. Data were gathered through interviews with the financial and administrative officials of the faculty, as well as an analysis of documents from the accounting and administrative departments. Data were collected using a researcher-developed checklist and analyzed with Microsoft Excel 2013 software.

In the ABC system, costs are assigned to activities based on the resources they consume in delivering services. The core principle is that services use activities, which in turn consume resources. Therefore, costs are first allocated to activities, and these activity-related costs are subsequently assigned to cost objectives using appropriate allocation bases. Costing is the process of determining the total cost of activities necessary for providing services or products.

In this study, after defining activity centers and conducting costing, the process of allocating costs from overhead activity centers to final activity centers (educational groups) was carried out. Suitable bases for distributing the costs of overhead and intermediate centers were established (Table 1). These bases facilitated the assignment of costs from each activity center to the final activity center. The specific costs for the educational groups were then estimated and added to the allocated costs. The portion of these combined costs attributed to each student within the educational group was calculated, and finally, the overall cost of educating each student was determined (14-16).

**Table 1:** Cost centers and apportionment base

Type of cost center	Activity centers	Apportionment base
Overhead	Head office	Employees + Students
	facilities and technical office	Area
	network room	Employees + Students
	warehouse and boiler room	Employees + Students
	water station	Employees + Students
	lobby and public space	Employees + Students
	prayer room	Employees + Students
	administrative affairs	Employees + Students
	electrical room	Area
	Guard unit	Employees + Students
	Accounting	Employees
	Supply Unit	Employees
	Security	Employees + Students
	information technology	Employees + Students
Intermediate	educational department	Students
	medical equipment	Students
	Admission	Employees + Students
	changing room and dining area	Employees + Students
	occupational health	Students
	library and study hall	Students
	suction room	Students
	compressor room	Students
	general practitioner	Students
Final	Radiology	Students
	Classes	Students
	CSR	Students
	plaster room	Students
	unit hall	Students
	Pathology	Students
	Diagnostics	Students
	restorative phantom	Students
	Darkroom	Students
	educational groups	Students

To calculate the full cost of training dental students at IUMS using the ABC system, the study followed six steps:

### Step One: Analyzing the Faculty's Activity Processes and Communication

In the first phase of the study, the operational processes of educational groups and their interactions with supporting activity centers—including both overhead and intermediate centers—were analyzed.

It also examined the faculty's financial, accounting, and administrative systems to ensure the feasibility of subsequent steps. The cost structure in the education sector differs significantly from that in the production and service sectors. In production, a substantial portion of costs is attributed to consumables, equipment, and capital goods, with labor costs often being minimal. In contrast, the education sector is heavily reliant on human resources, with a significant share of costs attributed to faculty members and staff (15, 16).

### Step Two: Identifying Activity Centers

A critical component of the ABC system is the accurate identification and definition of activity centers. To achieve this, the study first analyzed the various activities and referred to the organizational chart to identify all relevant activity centers, ranging from leadership to educational groups within the faculty. These centers are responsible for generating direct costs internally and for incurring indirect costs through interactions with other centers.

### Step Three: Classifying Activity Centers by Their Services and Outputs

Following the identification of activity centers, the study defined the services and outputs of each center, as well as the sequence of activities involved in delivering those services. The activity centers were classified into three primary groups based on their functions:

**Overhead Activity Centers**: These centers provide general and support services to operational (final) and intermediate activity centers. They are not directly involved in delivering services to students or employees. Their costs are considered overhead for other units.

**Intermediate Activity Centers**: These centers provide services to operational departments, students, and staff. They can be considered independent cost units. Centers that offer services to both intermediate and final activity centers, including educational groups, are classified as intermediate activity centers.

**Operational and Final Activity Centers**: These centers are directly involved in service delivery. In this study, the educational groups that provide teaching services to students are recognized as the operational and final activity centers. These centers receive support from both overhead and intermediate centers.

### Step Four: Identifying Resources and Performing Costing for Activity Centers

In this stage, after identifying and classifying the activity centers, data on resources and equipment related to each activity center were collected and assessed. This included information on building area, properties and equipment, human resources, consumables, and municipal services. Costing for each activity center was carried out based on the activity driver.

The costs identified from the resources were as follows:

**Building depreciation costs**: To calculate the depreciation costs of the building for each activity center, the construction cost per square meter in Tehran was first estimated. Using this information, the total construction cost was calculated with the data presented in Table 2(13, 15), applying the following formula:


$$\text{Building Depreciation Cost}=\frac{\text{Building area}\times \text{Cost per square meter of construction}}{\text{Useful life of the building}}$$

**Table 2:** Asset lifespan

Fixed Asset	Minimum lifespan (Year)	Maximum lifespan (Year)
High-quality building	50	100
Dilapidated and unstable building	15	50
Central facilities for steam and hot water generation	30	40
Elevators	20	30
Office furniture and equipment	10	20
Transportation vehicles and machinery	5	10
Medical electrical and mechanical equipment	10	20
Medical equipment for laboratory and surgical use	5	10
Non-electrical medical metal furniture	10	20
Stainless steel container	15	20
Laundry equipment	5	15
Workshop and firefighting equipment	10	15
Gardening tools	5	10

The depreciation cost for each unit was then estimated based on its proportionate share of the building.

**Depreciation costs for each unit**: The depreciation cost for each unit was estimated based on its proportionate share of the building.

**Equipment Depreciation Costs**: To calculate the total cost, annual depreciation for equipment and capital goods, such as desks, chairs, and computers, was determined using the straight-line accounting method. The annual depreciation cost is calculated as follows:


$$\text{Annual Depreciation}=\frac{\text{Total current value}-\text{Salvage value}}{\text{Useful life of capital goods}}$$

**Human resource costs**: Personnel costs for all activity centers, including salaries, benefits, insurance, taxes, deductions, etc., were collected and analyzed.

**Consumables and supplies costs**: These costs included expenses for printing, purchasing publications, ceremonial costs, postal charges, consumables for facilities and computer units, research expenses, and administrative office and kitchen supplies.

**Utility costs**: Utility expenses for water, electricity, gas, and telephone were collected from the accounting department and allocated to the units based on appropriate cost allocation bases.

By summing the costs of building depreciation, equipment, human resources, consumables, and utilities, the total internal costs for each activity center were calculated.

### Step Five: Allocating the Costs of Overhead and Intermediate Activity Centers to the Final Centers

In this study, the costs of each center were categorized into two groups: 1) specific costs within each activity center, and 2) costs allocated from other activity centers according to the allocation bases. The total cost of each activity center (TCi) includes both specific and overhead costs, calculated as follows:


$$TC_{i}=B_{i}PB+\sum n_{j}=E_{ij}PE_{ij}+\sum n_{j}=L_{ij}W_{ij}+\sum n_{j}=C_{ij}PC_{ij}+U_{i}PU+OC_{i}$$

Where:

TCi = Total cost of activity center i
Bi = Space occupied by center iii
PB = Depreciation cost per square meter of the building
Eij = Number of equipment units in group j in activity center i
PEij = Depreciation cost of equipment j in activity center i
Lij = Number of personnel in group j in activity center i
Wij = Wages and benefits of personnel in group j in activity center i
Cij = Number of consumable units in group j in activity center i
PCij = Price per unit of consumable j
Ui = Units of utilities (e.g., water, electricity, gas, telephone) consumed by activity center iii
PU = Price of utilities
OCi = Other costs associated with activity center i

In the cost allocation process, overhead activity centers serve as the starting point for costing and cost allocation operations. The costs from both overhead and intermediate activity centers were assigned to the final activity centers using appropriate allocation bases. After calculating the total costs for each center, the overhead cost share from other centers was estimated by dividing the respective costs by their allocation base values. This process was repeated for each of the overhead and intermediate centers individually. The share of overhead costs assigned to the educational groups (final activity centers) represents the cumulative cost shares from all overhead and intermediate centers.

### Step Six: Calculating the Full Cost of Training a Student

Once the total overhead and specific costs for the final activity center were calculated, the number of students was determined. The share of each student in the overhead and specific costs was calculated, which was then multiplied by the number of years required for study. This approach allowed for the determination of the full cost of educating each student throughout the entire course of study in dentistry.

**Ethical considerations**: This study is derived from the research project with the Code of Ethics IR.IUMS.REC. 1402.774, conducted at the Vice President of International Affairs, Iran University of Medical Sciences, Tehran, Iran.

## Results

The estimated average total cost of training a general dentistry doctoral student in the 2022-23 academic year at Iran University of Medical Sciences was approximately $132,438 (PPP), with an annual cost of $33,038 (PPP) during the educational phase and $16,591 (PPP) during the clinical phase. The analysis, which was based on activity identification and organizational charts from the dental school, identified a total of 33 activity centers, including 14 overhead activity centers, 9 intermediate activity centers, and 10 final activity centers.

**Overhead activity centers**: These centers provide services to all other activity centers.

**Intermediate activity centers**: These centers provide services to both intermediate and final activity centers, including educational groups.

**Final (operational) activity centers**: These are the centers directly engaged in delivering services and training students. The educational groups, which provide teaching services to students, were recognized as the final activity centers, supported by overhead and intermediate centers.

The results of this analysis are presented in a table, where all costs are adjusted based on the World Bank's purchasing power parity (PPP) conversion factors for 2022 in current international dollars.

**Figure 1:**
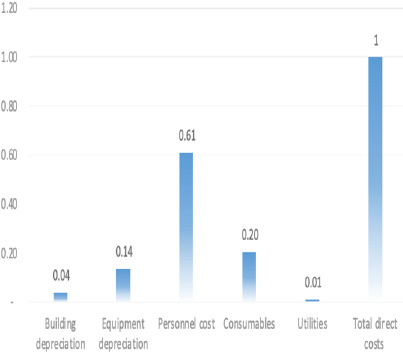
Cost-share of direct cost components

As illustrated in **Figure 1**, personnel costs represent the largest share of total costs, accounting for 61%, while utility costs constitute just 1%, the smallest share.

**Direct (specific) costs:** The direct (specific) costs associated with each activity center include depreciation of buildings and equipment, salaries and benefits of human resources, consumables, and utilities. The total direct costs for the final activity centers amounted to $1,133,853 (PPP), with theoretical courses accounting for $756,421 and practical courses for $377,432. Additionally, the total cost of the overhead activity centers was $802,953 (PPP). When combined, the total direct and overhead costs for the final activity centers reached $1,936,805 (PPP). Table 4 presents the breakdown of direct (specific) and overhead costs for both practical and theoretical courses.

**Table 4:** Overhead and Direct (Specific) Costs by Academic Course ($PPP)

Type of costs	Academic course	

	Theoretical	Practical
Overhead	234,721	568,231
Direct (specific)	756,421	377,432
Sum	991,143	945,663

Table 5 presents the average annual cost and the overall cost of educating a student in each academic phase, ultimately leading to the calculation of the total cost of training a general dentistry student.

**Table 5:** Cost of training a dentistry student ($PPP)

Dentistry discipline	Education level	Number of students	Average annual cost	Number of schooling years	Total cost of training	Average cost of training a student
Basic medical science course	Doctorate	30	33,038	2	66,076	132,438
Clinical course	Doctorate	57	16,591	4	66,362	

## Discussion

Dental education, traditionally structured into a four-year program requiring a significant number of staff for both theoretical and practical training, offers limited opportunities for improving the management of educational costs (2). It is suggested that when a dental school is effectively managed within its budget and aligns with the mission and objectives of the university, its costs become acceptable to the institution. Importantly, the value of a dental school should not be solely measured by its costs, but also by the quality of education, research, services, and the impact of graduates on community health (4).

Although the number of dental graduates continues to increase, evidence suggests that the proportion of the population who regularly visit dentists for routine treatments is decreasing. While more dentists are trained, patient treatment frequency has declined, with dentists compensating for this by raising fees. To address this issue, hospital-affiliated medical schools and medical groups are being considered as a solution. These institutions could train more students and increase graduate numbers at a lower cost through economies of scale, as expanding capacity does not necessarily double running costs (5).

Moreover, the cost of dental education should not merely be viewed as an expense but as an investment. This perspective requires assessing costs in terms of time, money, and the expected return on investment (8).

The present study estimates that the average cost of educating a general dentistry student at Iran University of Medical Sciences in the 2022-23 academic year is $132,438 (PPP). A significant portion of this cost (61%) is attributed to human resources, followed by consumables (20%) and equipment depreciation (14%). These findings indicate that human resource-related expenses are the major cost drivers in dental education. Therefore, financial planning aimed at improving education quality should focus on optimizing human resources to increase efficiency and reduce unnecessary costs.

In a similar study conducted by Pouragha and colleagues in 2020 at Alborz University of Medical Sciences, the average annual cost of services for each student was $4,778, with 65% of this cost dedicated to wages and salaries, 26% to depreciation of buildings and equipment, and 9% to consumables (17). Similarly, Rahimniya et al. (2011) estimated the cost of student services at Tehran University of Medical Sciences using ABC and found the full cost of services to be 12,465,916 rials (18).

A study conducted at Mazandaran University revealed that the average cost per student in the Dental and Pharmacy Schools was $8,582 and $5,789, respectively. Faculty salaries and benefits accounted for the highest costs (19). Doroodi et al. (2020) concluded that the total cost of educating each PhD and Master's student in the Health Management and Economics Sciences group was approximately 433 and 150 million rials, respectively, with 78% of the costs attributed to direct activity centers (15). Rezaei and associates (2022) estimated the cost of educating each medical student at AJA University of Medical Sciences to be 6,267,568,065 rials, noting that the underutilized capacity in overhead and intermediate centers was 30% and 33%, respectively (16). These findings are consistent with the results of this study when adjusted for inflation.

In a study by Tabriz University of Medical Sciences, personnel costs represented 74% of the total student training costs (20). Other studies, such as one by Namazi et al., showed that educational costs in general represent about 70% of total student education costs, with various factors such as the number of students per major, education level, and field of study influencing these costs (22).

The use of Activity-Based Costing (ABC) has proven useful in identifying the root causes of rising higher education costs. Given the high proportion of personnel costs, strategic planning around human resource management and addressing underutilized faculty members could improve efficiency and productivity (23). Furthermore, the growing demand for tools that assist in managerial decision-making, driven by increased competition among universities and regulatory requirements, highlights the need for clear, measurable cost-effectiveness indicators.

Universities, being among the largest public institutions, face unique challenges in cost recording due to the diversity of their activities. The increasing demand for detailed cost information makes the implementation of management support tools, such as ABC, essential (24). ABC helps track the costs associated with each activity based on its frequency or demand, leading to more accurate evaluations of budgetary issues and financial allocations, and ultimately improving financial management within universities (23).

Personnel costs, especially payments to faculty members, and capital expenditures are the largest financial burdens in higher education. A study has shown that the high cost of dental education places a substantial financial burden on students, particularly those from lower socioeconomic backgrounds, with a significant portion of these expenses allocated to accommodation costs (4). To mitigate these challenges, innovations such as digital delivery of theoretical courses and more efficient use of facilities could reduce dependence on shared resources, lower administrative costs, and minimize faculty salary expenses. The COVID-19 pandemic demonstrated the feasibility of remote learning, which can reduce costs associated with traditional in-person courses (25-27).

This study is limited to the cost analysis of educational activities only. Costs associated with human resources and equipment used in patient care and income-generating activities were not included. Additionally, it was not possible to separate depreciation costs for buildings and equipment associated with therapeutic services.

In conclusion, the study findings indicate that personnel costs represent the most significant portion of overall educational expenses. Improving human resource productivity should be a top priority for dental schools. Furthermore, consumable costs are notably high. By leveraging ABC, dental schools can gain a clearer understanding of cost patterns, enabling more effective financial management and resource allocation. Future research could explore the cost differences between theoretical and practical training, as well as the role of technology in reducing dental education costs.

## Figures and Tables

**Table 3:** Direct costs per cost center and share of overhead costs ($PPP)

Type of Centers	Activity centers	Depreciation cost of building	Depreciation cost of equipment	Personnel costs	Supplies	Utilities	Direct (specific) cost	Allocation base	Value of Allocation base	The overhead share of each unit to other units
	Head office	6,143	2,936	33,268	34,264	540	77,151	Employees + Students	137	563
	facilities and technical office	498	23,821	66,535	68,226	276	159,356	Area	3,450	46
	network room	111	2,527	-	8,566	8	11,211	Employees + Students	137	82
	warehouse and boiler room	2,767	686	66,535	17,132	431	87,552	Employees + Students	137	639
	water station	1,328	514	66,535	8,566	333	77,277	Employees + Students	137	564
Overhead	lobby and public space	34,287	1,194	-	4,283	2,336	42,100	Employees + Students	137	307
	prayer room	415	465	-	2,141	28	3,050	Employees + Students	137	22
	administrative affairs	415	1,234	33,268	25,271	149	60,338	Employees + Students	137	440
	electrical room	166	62	-	1,071	11	1,310	Area	3,450	0
	Guard unit	249	200,504	33,268	10,172	138	244,331	Employees + Students	137	1,783
	accounting	166	2,052	20,456	4,074	132	26,880	Employees	50	538
	Supply Unit	249	852	20,456	2,855	138	24,551	Employees	50	491
	security	166	310	61,368	6,309	375	68,527	Employees + Students	137	500
	information technology educational department	249	972	20,456	4,203	138	26,018	Employees + Students	137	190
		498	831	107,858	7,487	397	117,072	Students	87	1,346
Intermediate	medical equipment	249	268	20,456	13,741	138	34,852	Students	87	401
	Admission	692	1,139	81,823	3,993	531	88,179	Employees + Students	137	644
	changing room and dining area	2,491	2,015	-	2,496	170	7,171	Employees + Students	137	52
	occupational health	277	1,674	20,456	2,995	140	25,541	Students	87	294
	library and study hall	775	3,300	17,183	3,744	174	25,175	Students	87	289
	suction room	111	738	-	5,615	8	6,471	Students	87	74
	compressor room	111	465	-	9,359	8	9,942	Students	87	114
	general practitioner	830	208	53,647	7,487	662	62,834	Students	87	722
	radiology	3,016	5,845	34,367	7,693	448	51,369	Students	87	590
	Class	3,404	643	-	2,063	10,826	16,936	Students	87	195
	CSR	2,767	10,184	-	27,504	189	40,644	Students	87	467
	plaster room	1,273	1,697	-	6,188	87	9,246	Students	87	106
Final	unit hall	19,925	26,026	103,101	33,005	1,600	183,656	Students	87	2,111
	pathology	2,491	1,050	-	22,462	170	26,172	Students	87	301
	diagnostics	2,214	1,400	17,183	18,250	272	39,319	Students	87	452
	restorative phantom	2,491	11,173	-	8,251	170	22,085	Students	87	254
	darkroom	111	697	-	4,126	8	4,941	Students	87	57
	educational groups	4,538	20,456	600,762	111,604	2,125	739,485	Students	87	8,500
	Whole school	95,472	327,939	1,478,981	495,197	23,153	2,420,741			
